# Single-Use Fluidic Electrochemical Paper-Based Analytical Devices Fabricated by Pen Plotting and Screen-Printing for On-Site Rapid Voltammetric Monitoring of Pb(II) and Cd(II)

**DOI:** 10.3390/s21206908

**Published:** 2021-10-18

**Authors:** Dionysios Soulis, Maria Trachioti, Christos Kokkinos, Anastasios Economou, Mamas Prodromidis

**Affiliations:** 1 Department of Chemistry, National and Kapodistrian University of Athens, 15771 Athens, Greece; dsoulis1@gmail.com (D.S.); christok@chem.uoa.gr (C.K.); 2 Department of Chemistry, University of Ioannina, 45110 Ioannina, Greece; trachioti93@gmail.com (M.T.); mprodrom@uoi.gr (M.P.)

**Keywords:** paper-based devices, plotting, voltammetry, screen-printing, trace metals

## Abstract

This work reports the fabrication of integrated electrochemical fluidic paper-based analytical devices (ePADs) using a marker pen drawing and screen-printing. Electrodes were deposited on paper using screen-printing with conductive carbon ink. Then, the desired fluidic patterns were formed on the paper substrate by drawing with a commercial hydrophobic marker pen using an inexpensive computer-controlled x-y plotter. The working electrode was characterized by cyclic voltammetry and scanning electron microscopy. The analytical utility of the electrochemical PADs is demonstrated through electrochemical determination of Pb(II) and Cd(II) by anodic stripping voltammetry. For this purpose, the sample was mixed with a buffer solution and a Bi(III) solution, applied to the test zone of the PAD, the metals were preconcentrated as a bismuth alloy on the electrode surface and oxidized by applying an anodic potential scan. The proposed manufacturing approach enables the large-scale fabrication of fit-for-purpose disposable PADs at low cost which can be used for rapid on-site environmental monitoring.

## 1. Introduction

Heavy metals are toxic species that can accumulate in living organisms via the consumption of food and water, breathing, and absorption through the skin [1,2,3,4,5,6,7,8,9,10]. Heavy metals are considered one of the main sources of pollution in the environment [1,2,3,4,5,6,7,8,9,10]. Among them, lead and cadmium represent a major concern for public health due to their high toxicity even at low concentrations. Since heavy metal species usually exist at trace levels in different samples, sensitive spectroscopic approaches are considered the “golden standard” for their determination [11,12,13]. However, electroanalytical methods, and in particular stripping analysis, have also been widely used for trace metal quantification thanks to their remarkable sensitivity which is due to the preconcentration step of the target metals on the surface of the working electrode [14,15]. In addition, stripping analysis exhibits some additional advantages compared to optical techniques, such as inexpensive and portable instrumentation, low power requirements, and rapidity which increase its scope for field analysis.

The current requirements for on-site analysis had a great impact on the way towards the development of miniaturized, low-cost, integrated, and disposable analytical devices and sensors [16,17,18,19]. In particular, over the recent years, paper-based analytical devices (PADs) have gained increased popularity. The key features of paper as a functional substrate for the fabrication of analytical devices include [20,21]: flexibility and conformability; low thickness and lightness; absorbency and high surface-to-volume ratio; hydrophilicity and capillary action; chemical and biological inertness; disposability and biodegradability, and low cost and wide availability. As a result of recent advances, different types of PADs have been developed for application in various fields [22,23,24,25,26]. Among the multitude of existing PADs, those that employ electrochemical detection (so-called ePADs) have attracted considerable attention [27,28,29,30]. In the context of ePADs, the most critical steps in the fabrication procedure are the patterning of the hydrophobic barriers and the formation of electrodes. The most widely used methodologies for creating hydrophobic patterns on paper are wax printing, inkjet printing and lithography [27,28,29,30]. However, pen-on-paper methodologies using commercial permanent marker pens offer some important advantages: they do not require pre- or post-fabrication treatment (heating or curing), make use of low cost equipment and commercially available writing stationery, and provide greater flexibility in the selection of the functional material and fabrication conditions [31,32,33,34]. Regarding the formation of electrodes on PADs, many fabrication routes exist [27,28,29,30]; among these, screen-printing offers an attractive alternative due to its operational flexibility [35].

Several paper-based devices have been reported for heavy metal and metalloid determination by stripping analysis. Most of them involve multi-layered constructions such as: paper disks placed atop planar electrodes [36,37,38,39]; paper sheets attached on commercial 3-electrode screen-printed electrodes [40,41,42,43]; paper devices with externally attached electrodes [44,45]. On the contrary, only a handful of truly integrated ePADs have been developed for stripping analysis of heavy metals and metalloids fabricated by: laquer spraying/screen-printing [46]; wax melting/sputtering [47]; wax printing/screen-printing [48,49]; inkjet-printing/screen printing [50]. Among all the aforementioned paper-based sensors, only very few deal with simultaneous stripping determination of Pb(II) and Cd(II) [36,41,42,43] and are all based on modular designs. In this context, it is highly desirable to develop integrated, disposable, low-cost ePADs for the simultaneous determination of Pb(II) and Cd(II) by stripping analysis.

Therefore, in this work, we describe a fully integrated single-use ePAD that is fabricated by a combination of screen-printing and pen drawing. Initially, the electrochemical cell is formed by screen-printing of graphite ink on chromatographic paper. Then, the fluidic pattern is created by plotting with a hydrophobic marker pen. This manufacturing approach is simpler, less labor intensive, faster, and more cost-effective than existing fabrication approaches using ePADs intended for stripping analysis [46,47,48,49,50]. The analysis involves the addition of the sample mixed with a buffer solution and spiked with Bi(III) onto the device and simultaneous determination of Cd(II) and Pb(II) by anodic stripping voltammetry on the bismuth-coated working electrode. Bismuth is selected as a “green” alternative to mercury which enhances the sensitivity and presents low toxicity [51]. The study of the working conditions with the ePADs involved: the type of paper; type of pen; type of the graphite ink; selection of the metal film material; supporting electrolyte; concentration of the Bi(III) solution; deposition potential; deposition time; stripping mode, and stability of the reference electrode. Finally, the ePADs were applied to the analysis of a lake water sample.

## 2. Materials and Methods

### 2.1. Reagents

All the chemicals were of analytical grade and purchased from Merck (Darmstadt, Germany)). Doubly distilled water was used throughout. Stock solutions containing 10 and 100 mg L^−1^ of different metals (Bi(III), Cd(II), Pb(II), Sn(II), Zn(II), In(III), Cu(II), and Tl(I)) were prepared from 1000 mg L^−1^ standard solutions after appropriate dilution with water. A stock 2.0 mol L^−1^ acetate buffer (pH 4.5) was prepared from sodium acetate and hydrochloric acid.

The papers used were: Macherey-Nagel chromatography paper MN 261, Whatman No. 1 chromatography paper, and Whatman No. 42 filter paper. The marker pens that were investigated were commercial and purchased from a local stationery stop: Staedtler permanent Lumocolor waterproof 0.4 mm (s) black, the Edding 300 permanent marker water-resistant 1.5–3 mm, the Grand Paint Marker Olejowy paint marker GR-25 1.8 mm, the Edding 780 0.8 mm (black), and the BIC Marking Pro ultra-resistant permanent marker 1.1 mm. Graphite inks (Loctite EDAG PF 407A or Loctite EDAG 423SS) were a gift from Henkel Belgium.

### 2.2. Instrumentation and Signal Evaluation

Fluidic patterns with the desired shape were drawn using the open-access software Inkscape version 1.0.1 (Inkscape Project, https://inkscape.org/about/ (accessed on 10 October 2021)). The AxiDraw extension for Inkscape was used for controlling an AxiDraw desktop x-y plotter (Evil Mad Science LLC, Sunnyvale, CA, USA) connected to a PC via a USB interface.

A semi-automatic screen printer (E2, EKRA), polyester screens (77/195-48 PW, SEFAR PET 1500) and a 75 durometer polyurethane squeegee were used for screen-printing of the electrodes. 

A JSM-7401f field emission SEM (JEOL, Tokyo, Japan) was used for surface characterization of the electrodes. 

For electrochemical measurements, a Palmsens potentiostat, controlled by the PSTrace 5.5 software, was used (Palmsens, Houten, The Netherlands). All electrochemical data evaluation was performed with the PSTrace 5.5 utilities.

### 2.3. ePAD Fabrication

**Figure 1:**
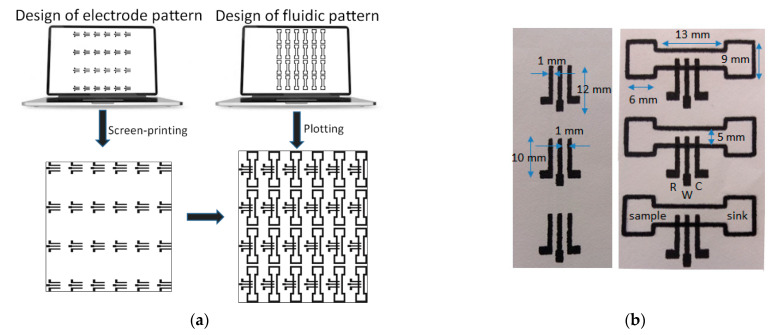
(**a**) Fabrication procedure of the ePADs, (**b**) screen-printed 3-electrode arrays (**left**), and fluidic ePADs (**right**). Three devices of each type are shown to illustrate the fabrication uniformity. The ePADs are composed of a sample zone, a sink zone, and the 3-electrode array (W, R, and C represent the working, reference, and counter electrode, respectively).

Fabrication of the ePADs proceeded in two steps (Figure 1a):

- Deposition of the three-electrode planar electrochemical cells. These were printed on paper sheets (Macherey-Nagel chromatography paper MN 261, thickness 180 μm) in 24 (4 × 6) arrays and consisted of graphite ink. Layers based the 423SS ink was cured after printing at 90 °C for 5 min using an infrared curing system (LittleRed-X2, VASTEX, Bethlehem, PA, USA). Layers based on the EDAG 407A ink were cured at 90 °C for 60 min in a conventional oven.
- Patterning of the PADs. The paper sheet with the arrays of the three-electrode cells was positioned onto a flat glass surface and aligned with the aid of pre-set alignment marks drawn from the sheets and the glass surface. The marker pen was inserted into the holder of the plotter and the 24 (4 × 6) PADs were drawn on the paper using a plotting speed of 0.76 cm s^−1^ and left at room temperature for 5 min to allow the solvent to evaporate. The pattern was repeated on the reverse side of the paper after aligning the paper. Finally, the paper was cut using scissors to obtain the individual ePADs which were carefully handled using tweezers. The nominal dimensions of the fluidic ePADs designed and fabricated in this work are shown in Figure 1b.

### 2.4. Experimental Procedure

For CV measurements, 100 μL of a 0.002 mol L^−1^ potassium ferrocyanide/0.002 mol L^−1^ potassium ferricyanide solution in 0.01 mol L^−1^ KCl was added in the sample zone of the device and the ePAD and allowed to flow past the three-electrode array. As soon as the solution reached the sink zone, the ePAD was connected to the potentiostat using crocodile clips and the CV was recorded in the range +1.V to −1.0 V at different scan rates.

For stripping voltammetry measurements, a 160 μL aliquot of the sample was spiked with 40 μL of the 2.5 mol L^−1^ acetate buffer (pH 4.5) solution and 2 μL of the 1000 mg L^−1^ Bi(III) standard solution. 100 μL of the mixed solution was placed at the sample zone of the ePAD and allowed to flow past the 3-electrode array. As soon as the solution reached the sink zone, the electrodes of the ePAD were connected to the potentiostat using crocodile clips, and accumulation was carried out at −2.5 V for 420 s vs. the carbon pseudo-reference electrode. Bismuth and the target metals were simultaneously reduced on the working electrode at −2.5 V according to the reactions:

Bi(III) + 3e^−^ → Bi

Pb(II) + 2e^−^ + Bi → Pb(Bi)

Cd(II) + 2e^−^ + Bi → Cd(Bi)

where Pb(Bi) and Cd(Bi) denote metal-bismuth alloys.

Then, a positive-going potential scan from −1.5 V to −0.2 V was applied in the differential-pulse (DP) mode (scan rate, 10 mV s^−1^; step, 5 mV; pulse height, 25 mV) or the square wave (SW) mode (frequency, 20 Hz; step, 2 mV; pulse height, 20 mV) and the voltammogram were recorded. This caused the metals and bismuth in the amalgam to oxidize, producing respective stripping peaks at distinct potentials:

Cd(Bi) → Cd(II) + 2e^−^

Pb(Bi) → Pb(II) + 2e^−^

Bi → Bi(III) + 3e^−^

All the measurements were carried out in the presence of dissolved oxygen.

## 3. Results

Electrochemical characterization of the ePADs was performed by cyclic voltammetry. CVs were recorded at different scan rates and the anodic and cathodic peaks currents were plotted as a function of the square root of the scan rate. The respective plots were highly linear suggesting diffusion-limited currents (Figure S1, Supplementary Materials).

**Figure 2:**
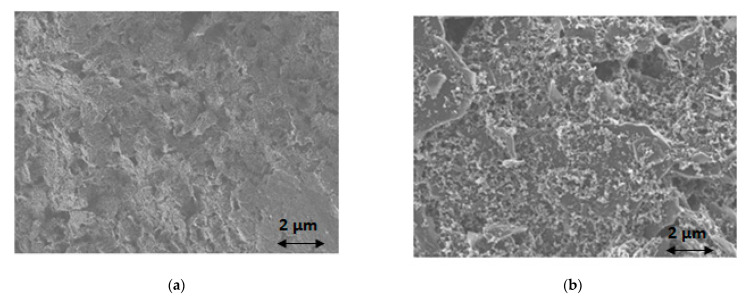
SEM images of (**a**) bare screen-printed working electrode surface; (**b**) bismuth-coated screen-printed working electrode surface.

For the microscopic study, coating of the working electrode’s surface with a bismuth film was performed by adding 100 μL of a 100 mg L^−1^ Bi(III) solution in 0.5 mol L^−1^ acetate buffer (pH 4.5) in the sample area of the ePAD and performing deposition at −2.0 V for 420 s after the solution reached the sink zone. Examination of the bare screen-printed working electrode’s surface with SEM reveals the rough surface of the conductive ink (Figure 2a). After coating with bismuth, the electrode surface is covered with bismuth nanoparticles (Figure 2b), suggesting successful deposition of bismuth on the surface of the screen-printed working electrode.

Among the 8 different papers used in our previous work involving pen plotting [34], the Macherey-Nagel chromatography paper MN 261, Whatman No. 1 chromatography paper, and Whatman No. 42 filter paper were shown to provide the best combination of transport characteristics and line consistency. In this work, the three types of paper provided statistically similar results, and the Macherey-Nagel chromatography paper MN 261 was used throughout.

Also based on our previous work [34], among the 17 marker pens tested for the formation of the fluidic channels, the following 5 pens provided satisfactory hydrophobicity for the isolation of the channels: the Staedtler permanent Lumocolor waterproof 0.4 mm (black), the Edding 300 permanent marker water-resistant 1.5–3 mm, the Grand Paint Marker Olejowy paint marker GR-25 1.8 mm, the Edding 780 0.8 mm (black) and the BIC Marking Pro ultra-resistant permanent marker 1.1 mm. The BIC Marking Pro ultra-resistant permanent marker 1.1 mm provides channel isolation with single-sided plotting while the other pens necessitate double-sided plotting. Unfortunately, spurious stripping peaks are obtained with the BIC Marking Pro ultra-resistant permanent marker 1.1 mm, probably to electroactive compounds leaching out of the particular ink. Among the remaining papers, the Edding 780 0.8 mm (black) with double-sided plotting was selected because it provided the best isolation of the channels in the acetate buffer used in this work; a plotting speed of 0.76 cm s^−1^ was selected.

Two types of graphite ink (Loctite EDAG PF 407A and Loctite EDAG 423SS) were tested for screen-printing under identical conditions. The latter produced the higher stripping peak for Pb and Cd and the best baseline.

**Figure 3:**
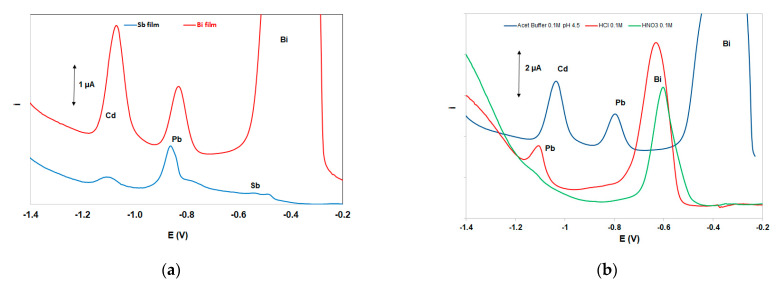
(**a**) Comparative DP voltammograms at a bismuth-film electrode and an antimony-film electrode, (**b**) comparative DP voltammograms in three supporting electrolytes. Conditions: 200 μg L^−1^ Pb(II) and Cd(II); supporting electrolyte 0.5 mol L^−1^ acetate buffer (pH 4.5) containing 10 mg L^−1^ Bi(III); deposition time, 420 s; deposition potential −2.5 V.

The two most widely used “green” metals (antimony and bismuth) were tested for the in situ formation of the metallic film on the working electrode [51]; both metals were added in the solution at 10 mg L^−1^. As illustrated in Figure 3a, the bismuth-film working electrode yielded the highest stripping signals for both Cd and Pb, and, therefore, bismuth was selected for the formation of the metal film. Under mildly acidic conditions (as used in this work) in situ-plated antimony, electrodes are known to produce weak stripping signals (probably due to gradual hydrolysis of Sb(III)) [52] and, thus, antimony electrodes are normally used in conjunction with strongly acidic media [51].

Three supporting electrolytes (0.5 mol L^−1^ of HCl, HNO_3_, and acetate buffer (pH 4.5)) were studied (Figure 3b). Only the sample in acetate buffer produced well-defined peaks for both Pb and Cd and this medium was elected as the supporting electrolyte. The strongly acidic HCl and HNO_3_ solutions produced higher background currents in the range of the Pb and Cd striping peaks (due to more pronounced proton reduction) which obscured the signals of the target metals.

**Figure 4:**
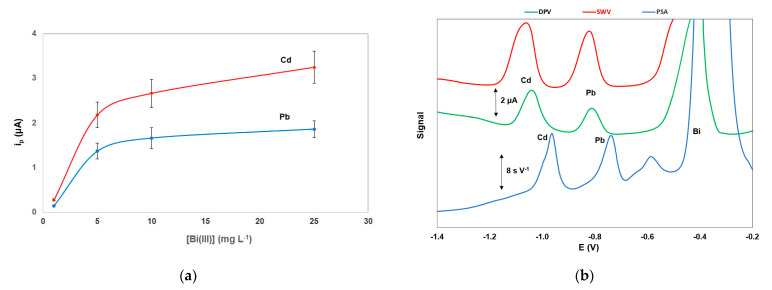
(**a**) Effect of the Bi(III) concentration on the stripping peak heights of Cd and Pb; (**b**) comparison between differential pulse voltammetry (DPV), square wave voltammetry (SWV), and constant current potentiometric stripping analysis (PSA). Conditions as in Figure 3.

The effect of the Bi(III) solution concentration used for the in-situ formation of the bismuth film on the stripping peak currents of Pb and Cd is illustrated in Figure 4a; the stripping signals for the two target metals increased with increasing Bi(III) concentration and remained almost constant for Bi(III) concentration higher than 10 mg L^−1^ which was selected for further work.

The deposition potential was studied in the range 0.0 to −3.0 V. The stripping signals of both the target metals increased as the deposition potential became more negative and leveled off at −2.5 V which was selected as the deposition potential (Figure S2a, Supplementary Material). The effect of the deposition time was investigated in the range 60–420 s and a linear dependence of the stripping signals of both the target metals vs. the deposition time was observed (Figure S2b, Supplementary Material); a deposition time of 420 s was selected.

Different stripping modes were investigated for the stripping step (Figure 4b). The constant-current potentiometric stripping analysis (PSA) mode produced a sloping baseline while the sensitivity and speed of the differential pulse (DP) waveform were lower than that of the square wave (SW) mode. However, the SW modulation caused widening of the peaks (in particular the Cd peak) which was more evident at higher metal concentrations.

In this work, a graphite carbon electrode was used as a quasi-reference electrode. Therefore, the peak potentials were highly dependent on the supporting electrolyte used (Figure 3b). However, in the selected 0.50 mol L^−1^ acetate buffer (pH 4.5) solution prepared from sodium acetate and hydrochloric acid (in order to achieve a constant Cl^−^ concentration which stabilized the potential of the pseudo-reference), peak potentials shifted by no more than ±10% between different ePADs.

**Figure 5:**
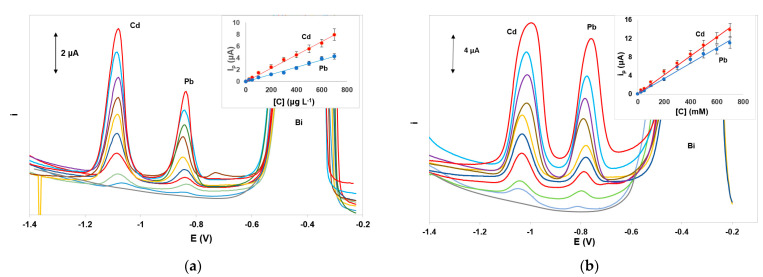
Voltammograms and respective calibration plots for the determination of Pb(II) and Cd(II) in the concentration range 0–700 μg L^−1^ by (**a**) DPASV and (**b**) SWASV. Conditions as in Figure 3.

Calibration for Cd(II) and Pb(II) was performed in the concentration range 0–700 μg L^−1^. The calibration features (calibration equation, coefficient of determination, and limits of quantification) for the target metals using DPASV and SWASV are summarized in Table 1. Under the selected conditions, the dynamic range for Pb(II) was 10.0–1000 μg L^−1^ nd for Cd(II) was 5.0–800 μg L^−1^. The limit of detection quantification (LOD) for each metal was calculated from the equation: LOD = 3.3 × s_b_/S (where s_b_ is the standard deviation of the intercept of the calibration plot and S is the slope of the calibration plot and the limit of quantification (LOQ) was calculated as: LOQ = 3 × LOD. Representative voltammograms and calibration plots using DPASV and SWASV are illustrated in Figure 5a,b, respectively. The analytical characteristics obtained with the integrated ePADS were comparable with those achieved with other non-integrated ePADs (Table S1, Supplementary Materials). Although these values are not as low as other more sophisticated bismuth-based sensors [51], they are considered adequate for single-use low-cost sensors intended for rapid on-site monitoring of heavy metals in environmental samples since they are lower than the legislative limits in the EU and the USA for drinking water (5.0 μg L^−1^ for Cd(II) and 10 or 15 μg L^−1^ for Pb(II) [53,54]).

The mechanical stability of the ePADs and the within-ePAD repeatability was studied by performing 5 consecutive stripping cycles (including sample addition, preconcentration, and stripping lasting in total 40 min) on a single ePAD; the % relative standard deviation was 5.3% for Pb(II) and 4.8% for Cd(II) while no solution leakage was observed. The between-ePAD reproducibility was calculated by performing six measurements with a solution containing 100 μg L^−1^ of the target metals at six different ePADs; the % relative standard deviations were 14.1% for Pb(II) and 13.5% for Cd(II). The mechanical stability of the ePADs was studied by performing 5 consecutive stripping cycles (including sample addition, preconcentration, and stripping) lasting in total 40 min.

The effect of common interfering cations (Sn(II), Sb(III), In(III), Tl(I), Zn(II), and Cu(II)) was studied. Sn(II), Zn(II), and Sb(III) did not interfere. Tl(I) and In(III) interfered severely by producing stripping peaks that overlapped with the Cd peak (Figure S3, Supporting Information); however, the concentration of these cations in the vast majority of typical samples is much lower than that of Cd(II). Cu(II) severely suppressed the peaks of both Pb and Cd and its interference was alleviated by the addition of ferrocyanide ions as suggested previously [55].

**Figure 6:**
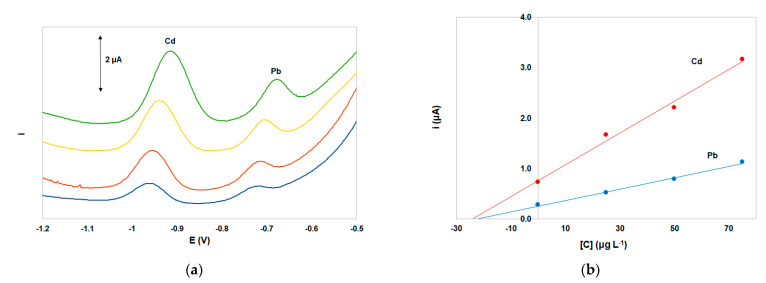
(**a**) SW voltammograms for the determination of Pb(II) and Cd(II) in a lake water sample spiked with 25 μg L^−1^ of Pb(II) and Cd(II), (**b**) respective standard addition plots. Supporting electrolyte 0.5 mol L^−1^ acetate buffer (pH 4.5) containing 10 mg L^−1^ Bi(III) and 1.0 × 10^−5^ mol L^−1^ K_4_[Fe(CN)_6_]. Other conditions as in Figure 3.

The ePADs were applied to Pb(II) and Cd(II) determination in a lake water sample and a tap water sample. In both samples, the Pb(II) and Cd(II) concentrations were lower than the limit of detection, and accuracy was determined by spiking and determination of the target metal concentrations by the method of standard additions. For the water sample, the recoveries were 97 ± 8% for Cd and 90 ± 9% for Pb (*n* = 3). Typical SW voltammograms and standard addition curves are illustrated in Figure 6. The slope for Pb(II) in the lake water sample was lower than the slope in the standard solutions and this was attributed to organic matter present in this particular sample; the slope of Cd(II) was not statistically altered by this effect. For the tap water sample, the recoveries were 98 ± 5% for Cd and 94 ± 6% for Pb (*n* = 3).

## 4. Conclusions

This work describes the fabrication of disposable integrated ePADs using a combination of pen plotting and screen printing. This manufacturing approach is cost effective, simple, and fast and enables large-scale fabrication of uniform devices. The ePADs were applied to the simultaneous determination of Pb(II) and Cd(II) by anodic stripping voltammetry using bismuth-coated working electrodes and the limits of detection were lower than the legislative limits. Detection requires only a portable potentiostat without any ancillary equipment (e.g., stirrers). In addition, no sample pre-treatment or solution deoxygenation is necessary. Therefore, the ePADS are fit-for-purpose concerning rapid on-site screening and monitoring of heavy metals in environmental samples.

## Figures and Tables

**Table 1:** Calibration features of the target metals at the ePADs.

Title 1	Cd(II)		Pb(II)	
	DP	SW	DP	SW
Slope ± SD (μA μg^−1^ L)	1.13 × 10^−2^ ± 2.3 × 10^−4^	1.96 × 10^−2^ ± 4.3 × 10^−4^	6.1 × 10^−3^ ± 2.3 × 10^−4^	1.63 × 10^−2^ ± 6.4 × 10^−4^
Intercept ± SD (μA)	2.0 × 10^−2^ ± 8.1 × 10^−3^	4.2 × 10^−2^ ± 1.6 × 10^−^^2^	5.5 × 10^−3^ ± 8.3 × 10^−3^	3.5 × 10^−2^ ± 2.1 × 10^−2^
R^2^	0.996	0.996	0.994	0.997
LOD (μg L^−1^) ^1^	2.4	2.7	4.5	4.2
LOQ (μg L^−1^) ^2^	7.1	8.1	13.5	12.8

^1^ Limit of detection. ^2^ Limit of quantification.

## Data Availability

Data is contained within the article or supplementary material.

## Supplementary Materials

The following are available online at www.mdpi.com/1424-8220/21/20/6908/s1, Figure S1: (α) CVs at the ePADs in 0.002 mol L^−1^ potassium ferrocyanide/0.002 mol L^−1^ potassium ferricyanide solution in 0.01 mol L^−1^ KCl; (b) plot of the anodic peak current (Ipa) and the cathodic peak current (Ipc) vs. the scan rate (U). Figure S2: Effect of the (a) deposition potential, (b) deposition time on the stripping peak heights of Pb and Cd. Table S1: Comparison of existing electrochemical paper-based devices for the determination of Pb(II) and Cd(II) by stripping analysis. Figure S3: Interference by (a) Tl(I), (b) In(III) on the SW stripping peaks of Pb and Cd.

## References

[1] Jarup J. (2003). Hazards of heavy metal contamination. Br. Med. Bull..

[2] Gupta V.K., Ali I., Aboul-Enein H.Y., Sarkar D., Datta R., Hannigan R. (2007). Metal ions speciation in the environment: Distribution, toxicities and analyses. Developments in Environmental Science.

[3] Masindi V., Muedi K., El-Din H., Saleh M., Aglan R.F. (2018). Environmental contamination by heavy metals. Heavy Metals.

[4] Chowdhury S., Mazumder M.A.J., Al-Attas O., Husain T. (2016). Heavy metals in drinking water: Occurrences, implications, and future needs in developing countries. Sci. Total Environ..

[5] Fernandez Luqueno F., Lopez Valdez F., Gamero Melo P., Luna S., Aguilera Gonzalez E.N., Martínez A.I., García M.S., Hernandez Martínez G., Herrera Mendoza R., Alvarez M.A. (2013). Heavy metal pollution in drinking water—A global risk for human health: A review. Afr. J. Environ. Sci. Technol..

[6] World Health Organization (1972). Evaluation of Certain Food Additives and the Contaminats Mercury, Lead, and Cadmium, Sixteenth Report of the Joint FAO/WHO Expert Committee on Food Additive.

[7] Mudgal V., Madaan N., Mudgal A., Singh R.B., Mishra S. (2014). Effect of toxic metals on human health. Open Nutraceuticals J..

[8] Azeh Engwa G., Udoka Ferdinand P., Nweke Nwalo F., Unachukwu M.N., Karcioglu O., Arslan B. (2019). Mechanism and health effects of heavy metal toxicity in humans. Poisoning in the Modern World-New Tricks for an Old Dog.

[9] QA M., MS K. (2016). Effect on human health due to drinking water contaminated with heavy metals. J. Pollut. Eff. Control.

[10] Chouhan B., Meena P., Poonar N. (2016). Effect of heavy metal ions in water on human health. Int. J. Sci. Eng. Res..

[11] Bhattacharjee T., Goswami M. (2018). Heavy metals (As, Cd & Pb) toxicity & detection of these metals in ground water sample: A review on different techniques. Int. J. Eng. Sci. Invent..

[12] Bulska E., Ruszczyńska A. (2017). Analytical techniques for trace element determination. Phys. Sci. Rev..

[13] Helaluddin AB M., Khalid R.S., Alaama M., Abbas S.A. (2016). Main analytical techniques used for elemental analysis in various matrices. J. Pharm. Res..

[14] Economou A., Kokkinos C., Arrigan D.W.M. (2016). Electrochemical Strategies in Detection Science.

[15] Borrill A.J., Reily N.E., Macpherson J.V. (2019). Addressing the practicalities of anodic stripping voltammetry for heavy metal detection: A tutorial review. Analyst.

[16] Thomas S., Ahmadi M., Nguyen T.A., Afkhami A., Madrakian T. (2021). Micro-and Nanotechnology Enabled Applications for Portable Miniaturized Analytical Systems.

[17] Rios A., Escarpa A., Simonet B. (2009). Miniaturization of Analytical Systems: Principles, Designs and Applications.

[18] Zhang W., Wang R., Luo F., Wang P., Lin Z. (2020). Miniaturized electrochemical sensors and their point-of-care applications. Chin. Chem. Lett..

[19] Tricoli A., Nasiri N., De S. (2017). Wearable and Miniaturized sensor technologies for personalized and preventive medicine. Adv. Funct. Mater..

[20] Nery E.W., Kubota L.T. (2013). Sensing approaches on paper-based devices: A review. Anal. Bioanal. Chem..

[21] Tribhuwan Singh A., Lantigua D., Meka A., Taing S., Pandher M., Camci-Unal G. (2018). Paper-based sensors: Emerging themes and applications. Sensors.

[22] Kung C.T., Hou C.Y., Wang Y.N., Fu L.M. (2019). Microfluidic paper-based analytical devices for environmental analysis of soil, air, ecology and river water. Sens. Actuat. B Chem..

[23] Fu L.M., Wang Y.N. (2018). Detection methods and applications of microfluidic paper-based analytical devices. Trends Anal. Chem. TrAC.

[24] Lim H., Turab Jafry A., Lee J. (2019). Fabrication, flow control, and applications of microfluidic paper-based analytical devices. Molecules.

[25] Ozer T., McMahon C., Henry C.S. (2020). Advances in paper-based analytical devices. Ann. Rev. Anal. Chem..

[26] Akyazi T., Basabe-Desmonts L., Benito-Lopez F. (2018). Review on microfluidic paper-based analytical devices towards commercialization. Anal. Chim. Acta.

[27] Noviana E., McCord C.P., Clark K.M., Jang I., Henry C.S. (2020). Electrochemical paper-based devices: Sensing approaches and progress toward practical applications. Lab. Chip.

[28] Ataide V.N., Mendes L.F., Gama L.I., de Araujo W.R., Paixao T.R. (2020). Electrochemical paper-based analytical devices: Ten years of development. Anal. Methods.

[29] Mazurkiewicz W., Podrażka M., Jarosińska E., Kappalakandy Valapil E., Wiloch M., Jönsson-Niedziółka M., Witkowska Nery E. (2020). Paper-based electrochemical sensors and how to make them (work). ChemElectroChem.

[30] Mettakoonpitak J., Boehle K., Nantaphol S., Teengam P., Adkins J.A., Srisa-Art M., Henry C.S. (2016). Electrochemistry on paper-based analytical devices: A review. Electroanalysis.

[31] Dossi N., Petrazzi S., Toniolo R., Tubaro F., Terzi F., Piccin E., Svigelj R., Bontempelli G. (2017). Digitally controlled procedure for assembling fully drawn paper-based electroanalytical platforms. Anal. Chem..

[32] Ghaderinezhad F., Amin R., Temirel M., Yenilmez B., Wentworth A., Tasoglu S. (2017). High-throughput rapid-prototyping of low-cost paper-based microfluidics. Sci. Rep..

[33] Amin R., Ghaderinezhad F., Li L., Lepowsky E., Yenilmez B., Knowlton S., Tasoglu S. (2017). Continuous-ink, multiplexed pen-plotter approach for low-cost, high-throughput fabrication of paper-based microfluidics. Anal. Chem.

[34] Pagkali V., Stavra E., Soulis D., Ecomomou A. (2021). Development of a high-throughput low-cost approach for fabricating fully drawn paper-based analytical devices using commercial writing tools. Chemosensors.

[35] Costa-Rama E., Fernández-Abedul M.T. (2021). Paper-based screen-printed electrodes: A new generation of low-cost electroanalytical platforms. Biosensors.

[36] Zhu C.C., Bao N., Huo X.L. (2020). Paper-based electroanalytical devices for stripping analysis of lead and cadmium in children’s shoes. RSC Adv..

[37] Feng Q.M., Zhang Q., Shi C.G., Xu J.J., Bao N., Gu H.Y. (2013). Using nanostructured conductive carbon tape modified with bismuth as the disposable working electrode for stripping analysis in paper-based analytical devices. Talanta.

[38] Bi X.M., Wang H.R., Ge L.Q., Zhou D.M., Xu J.Z., Gu H.Y., Bao N. (2018). Gold-coated nanostructured carbon tape for rapid electrochemical detection of cadmium in rice with in situ electrodeposition of bismuthin paper-based analytical devices. Sens. Actuat. B.

[39] Pokpas K., Jahed N., Iwuoha E. (2019). Tuneable, pre-stored paper-based electrochemical cells (μPECs): An adsorptive stripping voltammetric approach to metal analysis. Electrocatalysis.

[40] Nunez-Bajo E., Blanco-Lopez M.C., Costa-García A., Fernandez-Abedul M.T. (2017). Electrogeneration of gold nanoparticles on porous-carbon paper-based electrodes and application to inorganic arsenic analysis in white wines by chronoamperometric stripping. Anal. Chem..

[41] Sánchez-Calvo A., Blanco-López M.C., Costa-García A. (2020). Paper-based working electrodes coated with mercury or bismuth films for heavy metals determination. Biosensors.

[42] Ninwong B., Ratnarathorn N., Henry C.S., Mace C.R., Dungchai W. (2020). Dual sample preconcentration for simultaneous quantification of metal ions using electrochemical and colorimetric assays. ACS Sens..

[43] Thangphatthanarungruang J., Lomae A., Chailapakul O., Chaiyo S., Siangproh W. (2021). A low-cost paper-based diamond electrode for trace copper analysis at on-site environmental area. Electroanalysis.

[44] Pungjunun K., Chaiyo S., Jantrahong I., Nantaphol S., Siangproh W., Chailapakul O. (2018). Anodic stripping voltammetric determination of total arsenic using a gold nanoparticle-modified boron-doped diamond electrode on a paper-based device. Microchim. Acta.

[45] Nurak T., Praphairaksit N., Chailapakul O. (2013). Fabrication of paper-baseddevices by lacquer spraying method for the determination of nickel (II) ion in wastewater. Talanta.

[46] Mettakoonpitak J., Volckens J., Henry C.S. (2020). Janus electrochemical paper-based analytical devices for metals detection in aerosol samples. Anal. Chem..

[47] Wang X., Sun J., Tong J., Guan X., Bian C., Xia S. (2018). Paper-based sensor chip for heavy metal ion detection by SWSV. Micromachines.

[48] Pungjunun K., Nantaphol S., Praphairaksit N., Siangproh W., Chaiyo S., Chailapakul O. (2020). Enhanced sensitivity and separation for simultaneous determination of tin and lead using paper-based sensors combined with a portable potentiostat. Sens. Actuat. B Chem..

[49] Cinti S., De Lellis B., Moscon D., Arduini F. (2017). Sustainable monitoring of Zn (II) in biological fluids using office paper. Sens. Actuat. B.

[50] Pokpas K., Jahed N., McDonald E., Bezuidenhout P., Smith S., Land K., Iwuoha I. (2020). Graphene-AuNP enhanced inkjet-printed silver nanoparticle paper electrodes for the detection of nickel (II)-Dimethylglyoxime [Ni (DMGH_2_)] complexes by adsorptive cathodic stripping voltammetry (AdCSV). Electroanalysis.

[51] Ariño C., Serrano N., Díaz-Cruz J.M., Esteban M. (2017). Voltammetric determination of metal ions beyond mercury electrodes. A review. Anal. Chim. Acta.

[52] Czop E., Economou A., Bobrowski A. (2011). A study of in situ plated tin-film electrodes for the determination of trace metals by means of square-wave anodic stripping voltammetry. Electrochim. Acta.

[53] The Drinking Water Directive. https://ec.europa.eu/environment/water/water-drink/legislation_en.html.

[54] Safe Drinking Water Act. https://www.epa.gov/sdwa.

[55] Kokkinos C., Raptis I., Economou A., Speliotis T. (2010). Determination of trace Tl (I) by anodic stripping voltammetry on novel disposable microfabricated bismuth-film sensors. Electroanalysis.

