# Supplementary material for: Single-Use Fluidic Electrochemical Paper-Based Analytical Devices Fabricated by Pen Plotting and Screen-Printing for On-Site Rapid Voltammetric Monitoring of Pb(II) and Cd(II)
Source: Sensors (Basel). 2021 Oct 18;21(20):6908. doi: 10.3390/s21206908 (PMC8539493; doi:10.3390/s21206908)
Supplement: Supplementary file 1 [file sensors-21-06908-s001.zip › sensors-1424223-supplementary.pdf]

Supplementary Material

Single-use fluidic electrochemical paper-based analytical devices fabricated by pen plotting and screen-printing for on-site rapid voltammetric monitoring of Pb(II) and Cd(II)

Dionysios Soulis [1], Maria Trachioti [2], Anastasios Economou [1,], Christos Kokkinos, Mamas Prodromidis*

[1] Department of Chemistry, National and Kapodistrian University of Athens, Athens, 157 71, Greece

[2] Department of Chemistry, University of Ioannina, Ioannina, 45 110, Greece

* e-mail: aeconomo@chem.uoa.gr; fax: +30 210 7274750; tel: +30 210 7274298

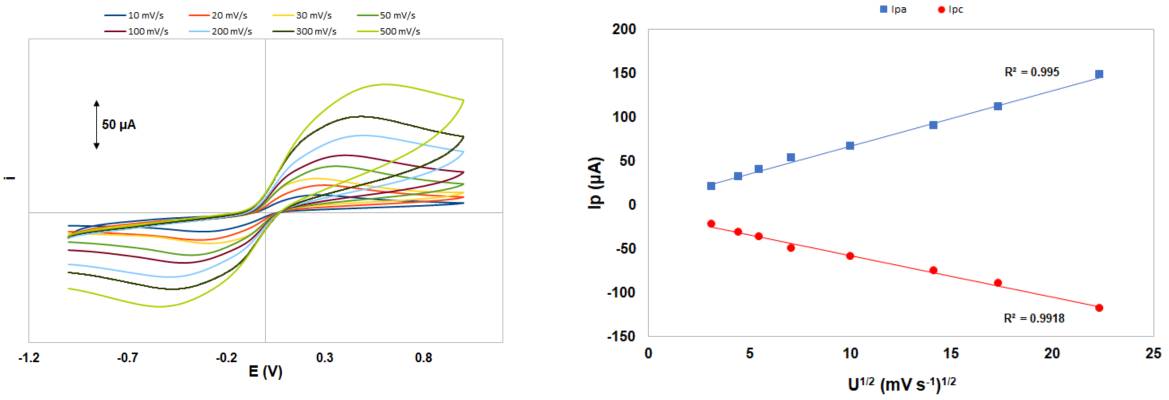

(a) (b)

Figure S1. (a) CVs at the ePADs in 0.002 mol L^{-1} potassium ferrocyanide/ 0.002 mol L^{-1} potassium ferricyanide solution in 0.01 mol L^{-1} KCl, (b) Plot of the anodic peak current (Ipa) and the cathodic peak current (Ipc) vs the scan rate (U).

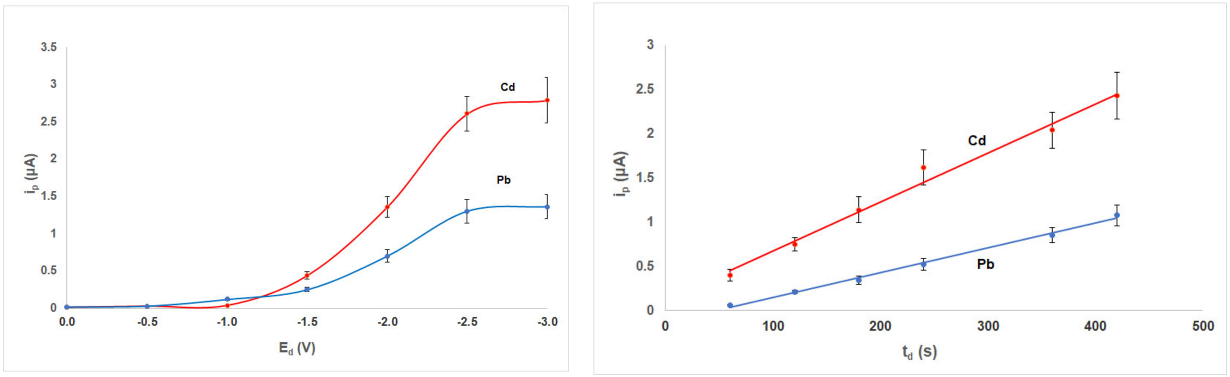

(a) (b)

Figure S2. Effect of the (a) deposition potential, (b) deposition time on the stripping peak heights of Pb and Cd. Conditions: 200 µg L^{-1} Pb(II) and Cd(II); supporting electrolyte 0.5 mol L^{-1} acetate buffer (pH 4.5) containing 10 mg L^{-1} Bi(III); deposition time, 420 s; deposition potential -2.5 V.

Table S1. Comparison of existing electrochemical paper-based devices for the determination of Pb(II) and Cd(II) by stripping analysis (in grayscale are the applications dealing with simultaneous determination of the two target metals).

**Type of Device**	**LOD (μg L^{-1})**		**Linear range (μg L^{-1})**		**Ref.**
	**Pb(II)**	**Cd(II)**	**Pb(II)**	**Cd(II)**	
Non-integrated	700[1]	1000[1]	1000-10000[1]	2500-10000[1]	41
	100[2]	400[2]	500-10000[2]	500-10000[2]	
Non-integrated	1	5	NR	NR	36
Non-integrated	2	-	NR	NR	37
Non-integrated	1	2.4	NR	NR	42
Non-integrated	0.5	0.5	0.5-400	0.5-400	43
Integrated	0.5	-	10–250	-	44
Integrated	4.2	2.4	10-1000	5-800	This work

[1] Bismuth-film

[2] Mercury-film

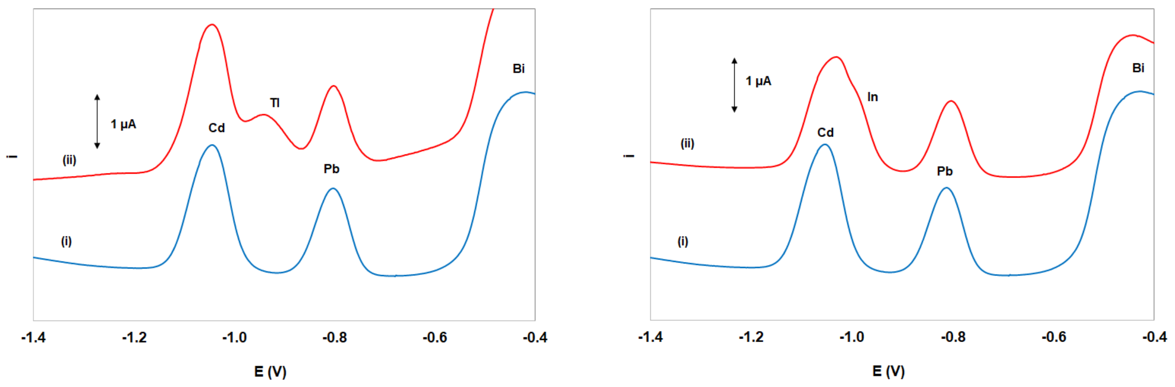

(a) (b)

Figure S3. Interference by (a) Tl(I), (b) In(III) on the SW stripping peaks of Pb and Cd. (i) Response without interferents, (ii) Response in the presence of interferennts. Conditions: 200 μg L^{-1} Pb(II), Cd(II), In(III) and Tl(I); supporting electrolyte 0.5 mol L^{-1} acetate buffer (pH 4.5) containing 10 mg L^{-1} Bi(III); deposition time, 420 s; deposition potential -2.5 V.
